# Stressful and potentially traumatic events and healthcare utilization: the 7^th^ Tromsø survey

**DOI:** 10.1186/s12913-025-12604-0

**Published:** 2025-03-27

**Authors:** Jens C. Thimm, Kamilla Rognmo, Ingunn Skre, Catharina E. A. Wang

**Affiliations:** 1 Department of Psychology, UiT The Arctic University of Norway, Tromsø, Norway; 2 Centre for Crisis Psychology, University of Bergen, Bergen, Norway

**Keywords:** Stressful life events, Potentially traumatic events, Healthcare utilization, Mental health, Population-based study

## Abstract

**Background:**

Stressful and potentially traumatic life events (SLEs/PTEs) can have a profound negative impact on the individual’s mental and physical wellbeing and health. Consequently, an association of SLEs/PTEs with increased healthcare utilization has been found. However, most studies have been conducted in selected samples (e.g., veterans), and there is a paucity of studies in the general population. The present study examined the associations between SLEs/PTEs and the utilization of healthcare services in the general population using data from the seventh survey of the Tromsø study (Tromsø7).

**Methods:**

The sample comprised 20,069 participants aged 40 years and above (52.5% female, mean age 57.3 years, *SD* = 11.4 years) who completed measures of SLE/PTE exposure in childhood/adolescence and adulthood (including a question about mental preoccupation with SLEs/PTEs), utilization of a variety of healthcare services (general practitioner, medical specialist, hospital, emergency room, mental health services, physiotherapist, and complementary and alternative medicine provider) in the previous year, and self-reported feeling of being anxious or depressed.

**Results:**

The results showed that SLE/PTE exposure is associated with an increased use of all healthcare services, especially mental health professionals. Exposure to physical and emotional neglect in childhood/adolescence, violence, and sexual abuse showed the strongest associations with the utilization of mental health services. The strength of the associations with health service utilization increased with the number of SLEs/PTEs. Finally, mental preoccupation with the event(s) moderated the associations between SLE/PTE exposure and the utilization of healthcare services but not self-reported feeling of being anxious or depressed.

**Conclusion:**

It is concluded that the prevention of SLEs/PTEs and screening for SLE/PTE exposure in healthcare services to provide trauma-informed care should be a prioritized public health focus.

## Introduction

It is widely recognized that adverse life events, such as exposure to physical and emotional neglect in childhood/adolescence, violence, sexual abuse, accidents, a life-threatening illness, or painful medical treatment (stressful life events/potentially traumatic events, SLEs/PTEs), can have a profound negative impact on an individual’s mental and physical health in the short term and over the life course [1]. In addition, SLE/PTE exposure in childhood is associated with poorer social functioning and lower socio-economic status later in life [2]. International studies suggest that most individuals (approx. 70%) will experience one or more SLEs/PTEs during their lifetime, with some variation between countries [3, 4].

SLE/PTE exposure is associated with a broad spectrum of unfavorable mental health outcomes, including, but not limited to, anxiety and depression [5–7]. Moreover, SLEs/PTEs in childhood are associated with an earlier onset, a chronic course, and relapse of mental disorders [8–10]. Exposure to SLEs/PTEs has also been linked to a variety of somatic disorders and health conditions, including, but not limited to, cardiovascular diseases, metabolic diseases, respiratory diseases, autoimmune diseases, neurological diseases, chronic pain, and medically unexplained symptoms [11–15]. A dose-response relationship is consistently found, with an increasing number of SLEs/PTEs being associated with a higher risk of negative mental and somatic health outcomes (e.g., [16, 17]). Biologically, hypothalamic-pituitary-adrenal axis hyperactivity and subsequent dysregulated physiological processes, including the immune system, and altered brain structure and connectivity are assumed to cause somatic diseases as well as psychopathology after SLE/PTE exposure [18–20]. Proposed psychological mechanisms include threat-focused information processing (e.g., attention bias and negative appraisals of the event) and emotion-related changes (e.g., reactivity, awareness, and regulation difficulties) [21, 22].

Based on the detrimental effects that SLEs/PTEs can have on mental and physical health, it can be expected that SLE/PTE exposure is associated with an increased use of healthcare services. Indeed, existing research evidence suggests that SLE/PTE exposed individuals use healthcare services to a higher degree than non-exposed individuals. For example, SLE/PTE exposure has been connected with a higher number of general practitioner (GP) visits, contact with mental health professionals, use of emergency room, and hospital stays (e.g., [23–27]). Moreover, a cumulative effect of SLEs/PTEs on healthcare utilization has been found (e.g., [23, 25, 27]). Further, SLE/PTE exposure has been shown to be associated with higher prescription rates of prescription drugs [24, 28], including psychotropic medication [29]. At the same time, individuals with a history of SLEs/PTEs have shown higher rates of late or cancelled appointments and no-show [30], poorer adherence to cardiovascular treatment [31] and reduced response to antidepressants [32–34]. Study findings suggest that the severity of the psychological burden caused by SLEs/PTEs moderates the use of healthcare services [24, 26, 35–38]. Thus, examining the associations between SLEs/PTEs and healthcare utilization provides insights into the mental health and physical disease burden connected with SLE/PTE exposure, as well as the economic costs for individuals and society for healthcare that can be attributed to SLEs/PTEs.

### The current study

Although available research findings suggest links between SLEs/PTEs and healthcare utilization, existing studies have important shortcomings. For example, most research on the associations between SLEs/PTEs and healthcare service utilization has been conducted in specific groups, e.g., veterans [39–41], refugees [42], or individuals exposed to specific events [43, 44], which may not be generalizable to the general population. In addition, the range of healthcare services that have been investigated has been limited. Importantly, examining the moderating role of the psychological burden of SLEs/PTEs and mental health could have implications for public health and healthcare by suggesting strategies to reduce healthcare utilization after SLE/PTE exposure. Therefore, the primary aim of the present study was to investigate the associations between SLEs/PTEs in childhood/adolescence and adulthood with the utilization of a variety of healthcare services in the general population. A second goal of the study was to explore whether the psychological impact of SLE/PTE exposure in terms of preoccupation with the event(s) and self-reported feeling of being anxious or depressed moderates the relationship between SLEs/PTEs and healthcare utilization.

## Methods

### Participants

The present study used data collected in the seventh survey of the Tromsø Study (Tromsø7; [45]). The Tromsø study is a population-based health study in the municipality of Tromsø in Northern Norway that was started in 1974 to investigate cardiovascular risk factors in men but was extended in scope in subsequent surveys to include a wider range of clinical examinations and assessments [46]. In Tromsø7 (2015–2016), all inhabitants of the municipality of Tromsø aged 40 years and older were invited to participate (*N* = 32,591) [45]. The sample in Tromsø7 was restricted to inhabitants aged 40 and above due to concerns about low response rates in younger age groups, entailing the risk selection bias and threatening the generalizability of the results. The response rate in Tromsø7 was 64.7% (*N* = 21,083). Because 14 participants withdrew their consent that their data are used in research, the sample size for the present study was *N* = 21,069 (52.5% female), with a mean age of 57.3 years (SD = 11.4 years, range 40 to 99 years). Further demographic characteristics of the sample are presented in Table 1.

**Table 1:** Sample characteristics

Demographic	
	*M* (*SD*)
Age	57.3 years (11.4 years)
	N (%)
Female	11,063 (52.5%)
Ethnicity	
Norwegian	19,030 (91.7%)
Sami, Kven/Finnish, and other	1,718 (8.3%)
Education	
Primary/partly secondary education	4,795 (23.2%)
Upper secondary education	5,748 (27.8%)
Tertiary education (< 4 years)	4,005 (19.4%)
Tertiary education (≥ 4 years)	6,143 (29.7%)
Living with spouse	15,274 (76.8%)
	*M* (*SD*)
Level of feeling of being anxious or depressed	1.27 (0.57)

**Table 2:** Prevalence of SLE/PTE exposure and mental preoccupation in the sample

SLEs/PTEs	N (%)
SLEs/PTEs before age 18	
Serious illness/accident	1,095 (5.3%)
Violence	1,213 (5.9%)
Sexual abuse	1,500 (7.3%)
Bullying	3,314 (16.2%)
Witnessed violence or sexual abuse	901 (4.4%)
Something else frightening	612 (3.0%)
Painful/frightening hospital treatment	713 (3.5%)
Painful/frightening dental treatment	3,833 (18.8%)
Illness/accident of a loved one	889 (4.4%)
Physical and emotional neglect	1,415 (6.9%)
SLEs/PTEs after age 18	
Serious illness/accident	3,650 (17.8%)
Violence	2,121 (10.3%)
Sexual abuse	592 (2.9%)
Bullying	1,167 (5.7%)
Witnessed violence or sexual abuse	1,039 (5.1%)
Something else frightening	1,058 (5.2%)
Painful/frightening hospital treatment	1,409 (6.9%)
Painful/frightening dental treatment	1,200 (5.9%)
Illness/accident of a loved one	5,977 (29.4%)
Still thinking a lot about what happened	886 (7.3%)

The current study was approved by the Regional Committee of Medical and Health Research Ethics (ref. 477677). The Norwegian Data Protection Service was notified about the study (ref. 755549).

### Measures

Questions about demographic information and the study measures used in the present investigation were included in the short general questionnaire (Q1) and the long general questionnaire (Q2) in the Tromsø7 study. Q1 could be answered on paper or online, whereas Q2 was administered online [45]. Q1 and Q2 (including English translations) are available from the webpage of the Tromsø study [47].

#### SLEs/PTEs

In Q2, a list of eleven SLE/PTE categories was included, developed for Tromsø7 to assess a broad range of SLEs/PTEs with a relatively small number of items to reduce the burden on the participants when responding to the comprehensive Q2 [4]. Participants were asked whether they have ever experienced one of the following events: (1) A life-threatening illness or a serious accident; (2) Violence; (3) Sexual abuse; (4) Bullying; (5) Witnessed someone close being exposed to violence or sexual abuse; (6) Experienced something else that was frightening, dangerous, or violent (for example, natural disaster, war, terror attack, held captive); (7) Death of a close one and difficulty accepting the loss, yearning for the deceased, and intense emotional pain related to the loss; (8) Received painful or frightening medical treatment when in hospital; (9) Received painful or frightening medical treatment at the dentist; (10) Someone close had a life-threatening illness or was exposed to a serious accident; and 11) Physical and emotional neglect in childhood [4]. The relationships of the loss of a loved one and severe grief with healthcare utilization are reported in a previous publication [48], and this SLE/PTE was therefore excluded from the present investigation. Four response options were provided: “No”, “Yes, before age 18”, “Yes, after age 18”, and “Yes, in the previous year”, except for childhood neglect, which could be answered with “No” or “Yes”. Because healthcare utilization during the previous year was assessed (see below), SLE/PTE exposure in the previous year was not included in the analyses in the present study to ensure that SLE/PTE exposure occurred before healthcare services were used. Mental preoccupation with SLEs/PTEs was measured with one question asking whether the participants still think a lot about what happened when they have experienced at least one of these events (response options: “No” and “Yes”).

#### Healthcare utilization

A list of healthcare providers was included in Q1 with the question whether the service was used in the previous year (response options: “No” and “Yes”). The following service providers were examined in the present investigation: general practitioner (GP), medical specialist (including hospital outpatient clinic), hospital, emergency room, mental health services (including psychologist/psychiatrist and psychiatric outpatient clinic), physiotherapist, and complementary and alternative medicine (CAM) provider (including acupuncturist and traditional healer). Participants were further asked to report the number of times they used the healthcare services in the previous year in a free-response format.

#### Anxiety and depression

Self-reported feeling of being anxious or depressed was assessed with one question in Q2, answered on a 5-point scale from 1 (“I am not anxious or depressed”) to 5 (“I am extremely anxious or depressed”).

### Statistical analyses

To examine the associations between SLE/PTE exposure with healthcare utilization in the previous year, a series of logistic regression analyses were performed with utilization of the seven healthcare services as the dependent variables and exposure to the assessed SLEs/PTEs before and after age 18 as predictors. Sex, age, ethnicity (Norwegian vs. non-Norwegian), and education level (dummy coded with primary/partly secondary education as reference category) were included as covariates in all analyses. First, all SLEs/PTEs in childhood/adolescence and adulthood were examined separately along with the covariates to predict the utilization of the seven healthcare services in the previous year. Further, the associations between the number of lifetime SLEs/PTEs reported with the utilization of the healthcare services in the previous year were investigated. The sum of a participant’s lifetime SLEs/PTEs was calculated when responses to at least eight of the ten SLEs/PTEs were provided (*N* = 20,435). Four categories were formed: no SLE/PTE exposure, one SLE/PTE, two SLEs/PTEs, and three or more SLEs/PTEs. The associations between the individual SLEs/PTEs and the number of times the different healthcare providers were used in the previous year were examined with negative binomial regression analyses. To ease the interpretation of the results of these analyses, standardized mean differences (Cohen’s d) were calculated as effect sizes. Cohen [49] suggested that *d*-values of 0.20, 0.50, and 0.80 indicate small, medium, and large effect sizes, respectively. Finally, separate moderation tests were run with lifetime SLE/PTE exposure as predictor, the use of any healthcare service in the previous year as dependent variable, and the variables thinking a lot about what happened and feeling of being anxious or depressed as moderators, respectively. No weighting for non-response was applied in the statistical analyses, and unweighted results are presented in the results section.

The analyses were conducted in R [50] (version 4.3.1). The misty package [51] (version 0.4.12) was used to calculate descriptive statistics. The MASS [52] (version 7.3–60) and countES packages [53] (version 0.0.0.9000) were used to perform negative binomial regression analyses and to calculate effect sizes, respectively.

## Results

Demographic characteristics of the sample (including information about the frequency of SLE/PTE exposure and the use of healthcare services) are displayed in Tables 1, 2 and 3. In total, 68.0% of the participants reported that they have experienced at least one of the examined SLEs/PTEs in childhood/adolescence or adulthood. The average number of lifetime SLEs/PTEs was 1.64. The most frequently SLE/PTE reported was a life-threatening illness or a serious accident of a loved one in adulthood (29.4%). The least frequently reported SLE/PTE was sexual abuse in adulthood (2.9%). Overall, 85.1% of the participants used at least one of the included healthcare services in the previous year, ranging from 4.2% (mental health service) to 80.5% (general practitioner).

**Table 3:** Healthcare utilization in the sample

Healthcare services	Used in the previous year *N* (%)	Mean number of times used in the previous year (SD)
General practitioner	16,841 (80.5%)	2.75 (3.51)
Medical specialist	8,059 (38.7%)	1.03 (3.03)
Hospital	2,292 (11.0%)	0.19 (1.13)
Emergency room	2,740 (13.5%)	0.18 (0.84)
Mental health service	863 (4.2%)	0.37 (3.21)
Physiotherapist	4,633 (22.7%)	2.14 (7.03)
CAM provider	2,102 (10.3%)	0.52 (2.82)

Note: *CAM* Complementary and alternative medicine

The bivariate associations between SLEs/PTEs in childhood/adolescence and adulthood and healthcare utilization in the previous year when controlled for age, sex, ethnicity, and education are displayed in Table 4. The results showed numerous statistically significant relationships. All SLEs/PTEs were significantly associated with the use of healthcare services in the previous year, and all included healthcare services showed an increased utilization after exposure to most kinds of SLEs/PTEs. For all SLEs/PTEs in childhood/adolescence, the highest odd ratios (*OR*s) were observed for the use of mental health services, ranging from 1.43 (95% CI 1.21 to 1.68) for painful or frightening dental treatment to 4.47 (95% CI 3.75 to 5.31) for physical and emotional neglect. Further, exposure to violence, sexual abuse, bullying, witnessing violence or sexual abuse, and physical and emotional neglect in childhood/adolescence were statistically significantly associated with the utilization of all seven assessed healthcare services in the previous year. The fewest associations (three) were observed for painful or frightening dental treatment. For SLEs/PTEs in adulthood, all SLEs/PTEs but one’s own or a loved one’s serious illness or accident were most strongly associated with the utilization of psychologist or psychiatrist in the previous year, with *OR*s ranging from 1.91 (95% CI 1.51 to 2.40) for painful or frightening dental treatment to 3.85 (95% CI 3.01 to 4.89) for sexual abuse. Own serious illness or accident showed the strongest association with the use of CAM provider (*OR* = 1.45, 95% CI 1.29 to 1.62). A serious illness or accident of a loved one was most strongly related to the utilization of a medical specialist (*OR* = 1.20, 95% CI 1.13 to 1.28). Adulthood exposure to a serious illness or accident, violence, sexual abuse, bullying, and painful or frightening hospital treatment was statistically significantly associated with the utilization of all included healthcare services in the previous year. The fewest statistically significant associations with the utilization of healthcare services (four) were found for a serious illness or accident of a loved one.

**Table 4:** Associations between individual SLEs/PTEs and use of healthcare providers (controlled for age, sex, ethnicity, and education)

	General practitioner	Medical specialist	Hospital	Emergency room	Mental health service	Physio-therapist	CAM provider
	*OR* (95% CI)	*OR *(95% CI)	*OR* (95% CI)	*OR* (95% CI)	*OR* (95% CI)	*OR* (95% CI)	*OR* (95% CI)
SLEs/PTEs before age 18							
Serious illness/accident	1.20 (1.02, 1.41)	1.26 (1.11, 1.43)	1.11 (0.91, 1.36)	1.14 (0.95, 1.35)	1.72 (1.33, 2.18)	1.22 (1.06, 1.41)	1.35 (1.12, 1.62)
Violence	1.42 (1.22, 1.67)	1.35 (1.20, 1.52)	1.45 (1.21, 1.74)	1.28 (1.08, 1.52)	2.96 (2.40, 3.62)	1.23 (1.07, 1.42)	1.67 (1.40, 1.98)
Sexual abuse	1.40 (1.20, 1.64)	1.33 (1.19, 1.48)	1.43 (1.21, 1.68)	1.47 (1.27, 1.70)	3.09 (2.57, 3.71)	1.19 (1.05, 1.34)	1.36 (1.16, 1.58)
Bullying	1.23 (1.11, 1.35)	1.21 (1.12, 1.31)	1.17 (1.03, 1.33)	1.24 (1.11, 1.38)	1.90 (1.61, 2.23)	1.13 (1.03, 1.23)	1.25 (1.11, 1.41)
Witnessed violence or sexual abuse	1.55 (1.29, 1.88)	1.19 (1.03, 1.37)	1.34 (1.07, 1.65)	1.29 (1.06, 1.56)	2.75 (2.18, 3.43)	1.22 (1.04, 1.42)	1.28 (1.05, 1.56)
Something else frightening	1.42 (1.09, 1.86)	1.28 (1.08, 1.52)	1.26 (0.99, 1.57)	1.45 (1.16, 1.79)	2.32 (1.64, 3.21)	1.14 (0.93, 1.39)	1.47 (1.13, 1.89)
Painful/frightening hospital treatment	1.38 (1.13, 1.71)	1.27 (1.09, 1.48)	1.24 (0.97, 1.56)	1.36 (1.10, 1.67)	2.37 (1.80, 3.07)	1.23 (1.03, 1.47)	1.33 (1.05, 1.66)
Painful/frightening dental treatment	1.09 (0.99, 1.20)	1.10 (1.02, 1.19)	1.02 (0.90, 1.14)	1.02 (0.92, 1.14)	1.43 (1.21, 1.68)	1.06 (0.97, 1.16)	1.15 (1.03, 1.29)
Illness/accident of a loved one	1.25 (1.05, 1.50)	1.19 (1.03, 1.37)	1.19 (0.95, 1.47)	1.28 (1.05, 1.54)	1.79 (1.36, 2.31)	0.98 (0.83, 1.15)	1.01 (0.81, 1.26)
Physical and emotional neglect	1.71 (1.46, 2.02)	1.33 (1.19, 1.49)	1.76 (1.50, 2.06)	1.33 (1.14, 1.55)	4.47 (3.75, 5.31)	1.35 (1.19, 1.52)	1.55 (1.32, 1.81)
SLEs/PTEs after age 18							
Serious illness/accident	1.33 (1.20, 1.47)	1.43 (1.32, 1.54)	1.25 (1.12, 1.40)	1.30 (1.17, 1.44)	1.42 (1.19, 1.69)	1.37 (1.25, 1.49)	1.45 (1.29, 1.62)
Violence	1.31 (1.17, 1.48)	1.21 (1.10, 1.33)	1.40 (1.21, 1.61)	1.41 (1.24, 1.60)	2.25 (1.87, 2.68)	1.38 (1.24, 1.53)	1.49 (1.30, 1.71)
Sexual abuse	1.73 (1.35, 2.27)	1.34 (1.13, 1.59)	2.00 (1.58, 2.51)	1.47 (1.16, 1.83)	3.85 (3.01, 4.89)	1.35 (1.12, 1.62)	1.66 (1.33, 2.06)
Bullying	1.77 (1.49, 2.12)	1.35 (1.20, 1.53)	1.59 (1.33, 1.89)	1.38 (1.17, 1.63)	2.68 (2.17, 3.28)	1.46 (1.28, 1.67)	1.45 (1.21, 1.72)
Witnessed violence or sexual abuse	1.55 (1.30, 1.85)	1.28 (1.12, 1.45)	1.14 (0.93, 1.40)	1.26 (1.05, 1.51)	2.03 (1.59, 2.56)	1.38 (1.20, 1.59)	1.32 (1.09, 1.59)
Something else frightening	1.05 (0.90, 1.23)	1.23 (1.08, 1.39)	1.11 (0.90, 1.35)	1.37 (1.15, 1.63)	2.11 (1.65, 2.68)	1.35 (1.16, 1.56)	1.50 (1.23, 1.80)
Painful/frightening hospital treatment	2.04 (1.72, 2.43)	1.79 (1.60, 2.00)	1.64 (1.40, 1.92)	1.73 (1.50, 1.99)	2.54 (2.08, 3.10)	1.67 (1.48, 1.89)	1.50 (1.28, 1.76)
Painful/frightening dental treatment	1.24 (1.06, 1.46)	1.23 (1.09, 1.39)	1.18 (0.98, 1.42)	1.38 (1.18, 1.62)	1.91 (1.51, 2.40)	1.27 (1.10, 1.45)	1.10 (0.91, 1.33)
Illness/accident of a loved one	1.12 (1.03, 1.21)	1.20 (1.13, 1.28)	1.00 (0.90, 1.11)	1.09 (0.99, 1.19)	1.02 (0.88, 1.19)	1.10 (1.02, 1.18)	1.16 (1.05, 1.29)

*CAM* Complementary and alternative medicine, *OR* Odds ratio. ORs with 95% confidence intervals that do not include 1 are underlined

The results of the analyses examining the relationship between the number of reported SLEs/PTEs with healthcare utilization are presented in Table 5. Overall, with an increasing number of SLEs/PTEs, the *OR*s for utilizing the different healthcare services also increased. Exposure to one SLE/PTE was significantly associated with the use of GP, medical specialist, physiotherapist, and CAM provider in the previous year. When three or more SLEs/PTEs were reported, the associations between SLEs/PTEs and the utilization of all healthcare providers were statistically significant. The highest *OR* was found for three or more lifetime SLEs/PTEs and mental health services utilization (*OR* = 3.81, 95% CI 3.13 to 4.66).

**Table 5:** Associations between the number of SLEs/PTEs and use of healthcare providers when controlled for demographics (age, sex, ethnicity, and education)

	General practitioner	Medical specialist	Hospital	Emergency room	Mental health service	Physio-therapist	CAM provider
Number of SLEs/PTEs							
One SLE/PTE vs no SLEs/PTEs	1.11 (1.01, 1.22)	1.21 (1.12, 1.30)	0.98 (0.87, 1.11)	1.12 (1.00, 1.25)	1.14 (0.89, 1.45)	1.11 (1.01, 1.22)	1.19 (1.04, 1.35)
Two SLEs/PTEs vs no SLEs/PTEs	1.22 (1.10, 1.36)	1.30 (1.19, 1.41)	1.05 (0.91, 1.20)	1.10 (0.96, 1.25)	1.47 (1.15, 1.88)	1.10 (0.99, 1.22)	1.28 (1.11, 1.47)
≥ Three SLEs/PTEs vs no SLEs/PTEs	1.66 (1.50, 1.83)	1.64 (1.52, 1.78)	1.36 (1.21, 1.54)	1.55 (1.38, 1.73)	3.81 (3.13, 4.66)	1.54 (1.41, 1.69)	1.78 (1.57, 2.02)

*CAM* Complementary and alternative medicine, *OR* Odds ratio. ORs with 95% confidence intervals that do not include 1 are underlined

The associations between SLEs/PTEs in childhood/adolescence and in adulthood and the frequency of utilization of the different healthcare providers in the previous year are shown in Table 6. Although most associations were statistically significant, the effect sizes were generally very small (*d* < 0.20) with only a few exceptions. The most associations with effect sizes greater or equal 0.20 were found for SLEs/PTEs and the number of mental health services visits (10 out of 19 effect sizes). The largest effect size was *d* = 0.67 (physical and emotional neglect in childhood and number of mental health services visits). Further, except for having experienced something else frightening and a life-threatening illness or serious accident of a loved one, all SLEs/PTEs in adulthood were positively related to the number of GP visits in the previous year with small to medium effect sizes, with painful hospital treatment showing the largest effect size (*d* = 0.43).

**Table 6:** Associations of SLEs/PTEs in childhood/adolescence and adulthood with the frequency of health service visits in the previous year (controlled for age, sex, ethnicity, and education)

	General practitioner		Medical specialist		Hospital		Emergency room		Mental health service		Physio-therapist		CAM provider	
	*b (SE)*	*d*	*b (SE)*	*d*	*b (SE)*	*d*	*b (SE)*	*d*	*b (SE)*	*d*	*b (SE)*	*d*	*b (SE)*	*d*
SLEs/PTEs before age 18														
Serious illness/accident	0.21^*^ (0.10)	0.06	0.11 (0.06)	0.05	0.42^***^ (0.11)	0.09	0.21^*^ (0.10)	0.06	0.73^*^ (0.30)	0.11	0.21 (0.12)	0.06	0.46^**^ (0.17)	0.11
Violence	0.24^**^ (0.09)	0.07	0.32^***^ (0.06)	0.16	0.52^***^ (0.11)	0.12	0.24^**^ (0.09)	0.07	1.17^***^ (0.29)	0.24	0.27^*^ (0.12)	0.08	0.51^**^ (0.17)	0.13
Sexual abuse	0.34^***^ (0.08)	0.11	0.22^***^ (0.06)	0.10	0.35^***^ (0.10)	0.08	0.34^***^ (0.08)	0.11	1.29^***^ (0.26)	0.28	0.28^*^ (0.11)	0.08	0.41^**^ (0.15)	0.09
Bullying	0.22^***^ (0.06)	0.06	0.13^**^ (0.04)	0.06	0.06 (0.07)	0.01	0.22^***^ (0.06)	0.06	0.85^***^ (0.18)	0.14	0.15 (0.08)	0.04	0.29^**^ (0.11)	0.06
Witnessed violence or sexual abuse	0.21^*^ (0.11)	0.06	0.14 (0.07)	0.06	0.34^**^ (0.13)	0.07	0.21^*^ (0.11)	0.06	1.25^***^ (0.33)	0.27	0.28^*^ (0.14)	0.08	0.45^*^ (0.19)	0.11
Something else frightening	0.27^*^ (0.13)	0.08	0.30^***^ (0.09)	0.15	0.24 (0.15)	0.05	0.27^*^ (0.13)	0.08	1.57^***^ (0.41)	0.41	0.11 (0.17)	0.03	0.10 (0.24)	0.02
Painful/frightening hospital treatment	0.33^**^ (0.11)	0.10	0.24^**^ (0.08)	0.11	0.04 (0.15)	0.01	0.33^**^ (0.11)	0.10	1.16^**^ (0.37)	0.23	0.45^**^ (0.15)	0.15	0.52^*^ (0.21)	0.13
Painful/frightening dental treatment	0.03 (0.06)	0.01	0.14^***^ (0.04)	0.06	0.02 (0.07)	0.00	0.03 (0.06)	0.01	0.38^*^ (0.17)	0.05	0.14^*^ (0.07)	0.04	0.30^**^ (0.10)	0.07
Illness/accident of a loved one	0.25^*^ (0.11)	0.08	0.24^***^ (0.07)	0.12	0.08 (0.13)	0.02	0.25^*^ (0.11)	0.08	0.74^*^ (0.33)	0.12	-0.13 (0.14)	-0.03	0.29 (0.19)	0.06
Physical and emotional neglect	0.21^*^ (0.10)	0.06	0.32^***^ (0.06)	0.16	0.55^***^ (0.10)	0.13	0.34^***^ (0.08)	0.11	1.96^***^ (0.26)	0.67	0.28^*^ (0.11)	0.08	0.57^***^ (0.15)	0.15
SLEs/PTEs after age 18														
Serious illness/accident	0.21^***^ (0.02)	0.22	0.34^***^ (0.04)	0.18	0.34^***^ (0.07)	0.08	0.24^***^ (0.06)	0.08	0.39^*^ (0.18)	0.05	0.47^***^ (0.07)	0.16	0.50^***^ (0.10)	0.12
Violence	0.26^***^ (0.02)	0.28	0.25^***^ (0.05)	0.12	0.27^**^ (0.09)	0.06	0.43^***^ (0.07)	0.14	1.05^***^ (0.22)	0.20	0.31^***^ (0.09)	0.10	0.58^***^ (0.13)	0.15
Sexual abuse	0.27^***^ (0.04)	0.30	0.42^***^ (0.08)	0.22	0.63^***^ (0.15)	0.16	0.36^**^ (0.13)	0.12	1.55^***^ (0.41)	0.40	0.28 (0.17)	0.08	0.49^*^ (0.23)	0.12
Bullying	0.35^***^ (0.03)	0.39	0.45^***^ (0.06)	0.24	0.57^***^ (0.11)	0.14	0.45^***^ (0.09)	0.28	1.43^***^ (0.29)	0.34	0.45^***^ (0.12)	0.15	0.34^*^ (0.17)	0.08
Witnessed violence or sexual abuse	0.26^***^ (0.03)	0.28	0.41^***^ (0.06)	0.21	0.46^***^ (0.12)	0.10	0.35^***^ (0.10)	0.11	0.74^*^ (0.31)	0.12	0.53^***^ (0.13)	0.18	0.25 (0.18)	0.05
Something else frightening	0.13^***^ (0.03)	0.13	0.23^***^ (0.06)	0.11	0.25^*^ (0.12)	0.05	0.29^**^ (0.10)	0.09	1.03^***^ (0.31)	0.19	0.38^**^ (0.13)	0.12	0.52^**^ (0.18)	0.13
Painful/frightening hospital treatment	0.38^***^ (0.03)	0.43	0.63^***^ (0.05)	0.37	0.58^***^ (0.10)	0.14	0.61^***^ (0.08)	0.22	1.20^***^ (0.27)	0.25	0.64^***^ (0.11)	0.23	0.74^***^ (0.15)	0.21
Painful/frightening dental treatment	0.21^***^ (0.03)	0.22	0.35^***^ (0.06)	0.18	0.48^***^ (0.11)	0.11	0.43^***^ (0.09)	0.14	0.79^**^ (0.29)	0.13	0.33^**^ (0.12)	0.10	0.19 (0.17)	0.04
Illness/accident of a loved one	0.09^***^ (0.02)	0.09	0.18^***^ (0.03)	0.08	0.11 (0.06)	0.02	0.05 (0.05)	0.01	0.11 (0.15)	0.01	0.15^*^ (0.06)	0.04	0.30^***^ (0.09)	0.06

^*^p < .05, ^**^p < .01, ^***^p < .001. *CAM* Complementary and alternative medicine

The results of the moderation analyses showed that the interaction of lifetime SLE/PTE exposure with thinking about what happened (*b* = 0.15, *z* = 2.25, *p* =.024) was statistically significant in the prediction of the utilization of any healthcare service in the previous year, but not the interaction between lifetime SLE/PTE exposure and the current level of anxiety and depression (*b* = −0.04, *z* = −1.36, *p* = .174).

## Discussion

In the present study, the associations between SLEs/PTEs and the use of a broad range of healthcare services, including GP, mental health professionals, and CAM provider, were examined in a large community sample of individuals aged 40 years and above.

Consistent with previous investigations into the associations between SLEs/PTEs and healthcare utilization in the general population [23, 26, 27], the present study found that reports of SLE/PTE exposure in childhood/adolescence and adulthood were related to an increased use of healthcare services in the previous year. Most SLEs/PTEs in childhood/adolescence and adulthood had their strongest associations with the use of mental health services in the previous year, especially the experience of physical and emotional neglect in childhood/adolescence, violence, and sexual abuse. In addition, painful or frightening hospital treatment and bullying in adulthood were among the SLEs/PTEs with the strongest associations with mental health service utilization in the previous year, suggesting a high mental burden that can be connected to these experiences [54–56]. Moreover, healthcare utilization associated with exposure to physical and emotional neglect in childhood, violence, bullying, and sexual abuse was not limited to mental health professionals but related to all healthcare providers assessed in the study, including medical services, physiotherapists, and CAM providers. However, in addition to the aforementioned SLEs/PTEs, the remaining SLEs/PTEs included in the survey (e.g., serious illness or accident of a loved one, painful or frightening hospital treatment) also showed significant associations with the utilization of several healthcare services. Although the associations were smaller in magnitude than for e.g. sexual abuse, some of these SLEs/PTEs are much more common in the population (e.g., serious illness or accident of a loved one), which increases their significance for healthcare utilization on a societal level [57, 58]. Further, consistent with previous research [24, 25], the study found that with an increasing number of SLEs/PTEs, the number of healthcare services that was used in the previous year and the strength of the associations with healthcare utilization increased, especially mental health services. This finding aligns with research reporting a cumulative effect of SLEs/PTEs on healthcare utilization [23, 25].

From an economic perspective, it is relevant to know not only which healthcare services that were used in connection with PTE/SLE exposure, but also the number of times the different healthcare providers are used. In line with previous studies [26, 36], SLEs/PTEs were in the present study significantly related also to the number of visits to healthcare providers in the previous year. The strongest association was between physical and emotional neglect in childhood and the number of mental health professional visits with a moderate to high effect size, indicating that the mental health problems connected with physical and emotional neglect in childhood require an especially high treatment intensity. This finding is in line with studies showing that childhood neglect is associated with an increased risk for mental disorders, more severe course, and poorer response to pharmacological and psychological treatment [59, 60]. Besides mental health service visits, the number of GP visits in the previous year emerged as a relatively strong correlate of SLE/PTE exposure. This finding can be due to the role of the GP in Norway. All registered inhabitants have the right to a GP, who functions as a gatekeeper for other public healthcare services.

Finally, the present study found that mental preoccupation with SLEs/PTEs, but not the self-reported feeling of being anxious or depressed, moderated the association between exposure to any SLE/PTE during lifetime and the utilization of any healthcare service in the previous year. Negative mental preoccupation with adverse experiences is related to the concepts of repetitive negative thinking and rumination, which have been shown to be concurrently and prospectively associated with emotional distress and psychopathology [61, 62]. Mental preoccupation can, however, also take a more functional form involving an assessment of one’s need for help leading to help-seeking behavior. There can therefore be different reasons why mental preoccupation moderated the associations between SLE/PTE exposure and healthcare service utilization in the previous year. Unfortunately, the wording of the item assessing mental preoccupation in Tromsø7 did not specify the affect and content of the preoccupation. On the other hand, self-reported feeling of being anxious or depressed did not emerge as a significant moderator of the relationships of SLEs/PTEs with healthcare utilization. In this study, self-reported feeling of being anxious or depressed was assessed with one question and as a state variable (feeling anxious or depressed at the time of the completion of the survey). It is possible that this simple and broad measure did not capture well the emotional burden associated with SLE/PTE exposure, resulting in the nonsignificant finding.

Taken together, the present study found that different types of lifetime SLEs/PTEs were statistically significantly associated with an increased utilization of a variety of healthcare services. These findings further imply that SLEs/PTEs are connected with considerable societal costs [63]. The strongest associations of SLEs/PTEs with healthcare utilization were found for exposure to physical and emotional childhood neglect, sexual abuse, and violence,, and multiple SLEs/PTEs with the utilization of a mental health services. Crucially, these SLEs/PTEs are preventable and should therefore be addressed in public measures. Most SLEs/PTEs were first and foremost associated with the utilization of mental health professionals. Previous research findings suggest, however, an underutilization of mental health services after SLE/PTE exposure due to a variety of reasons, including low mental health literacy, lack of access, concerns related to stigma, shame, rejection, and recalling painful memories, and financial costs [64]. In addition to mental health professionals, the experience of SLEs/PTEs was associated with the increased utilization of a number of other healthcare services. To better understand the health complaints of help-seeking patients and provide adequate, trauma-informed care, screening for SLEs/PTEs is recommended at these services [65].

Strengths of the present study are the large sample size and the broad range of SLEs/PTEs and healthcare services that were assessed. On the other hand, the study findings must be interpreted in the light of several limitations. First, SLE/PTE exposure and healthcare utilization were assessed using self-report that involves a risk of memory bias in contrast to registry-based data [66, 67]. Further, only persons aged 40 and older were invited to participate in Tromsø7, and the findings of the present study cannot, therefore, be generalized to younger age groups. It can be speculated that the associations between SLEs/PTEs and healthcare utilization are stronger in older age groups due to a potentially longer duration of psychological and physiological stress reactions after SLE/PTE exposure, negatively impacting mental and physical health, especially for events that occurred in childhood and adolescence. Moreover, the Tromsø7 sample is not representative of the population aged 40 and above in the municipality of Tromsø as especially the oldest age groups had low attendance rates [45]. Not weighting for non-response can have led to an overestimation of healthcare utilization in the population [68]. A final limitation of the present study is that healthcare utilization before SLE/PTE exposure was not assessed. Rosendal, Mortensen [69] found that healthcare utilization after a natural disaster was predicted by predisaster healthcare use rather than PTSD or PTSD symptoms. It is therefore possible that individuals with a high use of healthcare services have characteristics (e.g., coping styles) that predispose them to SLEs/PTEs and that SLE/PTE exposure itself is unrelated to healthcare utilization.

It can be concluded that self-reported SLE/PTE exposure is associated with an increased utilization of a variety of healthcare services, especially childhood neglect, violence, sexual abuse, bullying, and painful or frightening hospital treatment with the utilization of mental health services. Further, with the number of SLEs/PTEs, the associations with healthcare service utilization also increased. Mental preoccupation with the event(s) was associated with increased healthcare utilization. Prevention of SLEs/PTEs and screening for SLE/PTE exposure in healthcare services to provide adequate treatment should be a prioritized public health focus.

## Data Availability

The data that support the findings of this study are available from the Tromsø Study but restrictions apply to the availability of these data, which were used under license for the current study, and so are not publicly available.

## Abbreviations

CAM: Complementary and alternative medicine
GP: General practitioner
OR: Odds ratio
PTE: Potentially traumatic event
PTSD: Posttraumatic stress disorder
SLE: Stressful life event
